# PM10 promotes an inflammatory cytokine response that may impact SARS-CoV-2 replication *in vitro*

**DOI:** 10.3389/fimmu.2023.1161135

**Published:** 2023-04-25

**Authors:** Damariz Marín-Palma, Jorge H. Tabares-Guevara, María I. Zapata-Cardona, Wildeman Zapata-Builes, Natalia Taborda, Maria T. Rugeles, Juan C. Hernandez

**Affiliations:** ^1^ Infettare, Facultad de Medicina, Universidad Cooperativa de Colombia, Medellín, Colombia; ^2^ Grupo Inmunovirología, Facultad de Medicina, Universidad de Antioquia (UdeA), Medellín, Colombia; ^3^ Grupo de Investigaciones Biomédicas Uniremington, Programa de Medicina, Facultad de Ciencias de la Salud, Corporación Universitaria Remington, Medellín, Colombia

**Keywords:** particulate matter, SARS-CoV-2, inflammatory response, viral replication, air pollution

## Abstract

**Introduction:**

In the last decades, a decrease in air quality has been observed, mainly associated with anthropogenic activities. Air pollutants, including particulate matter (PM), have been associated with adverse effects on human health, such as exacerbation of respiratory diseases and infections. High levels of PM in the air have recently been associated with increased morbidity and mortality of COVID-19 in some regions of the world.

**Objective:**

To evaluate the effect of coarse particulate matter (PM10) on the inflammatory response and viral replication triggered by SARS-CoV-2 using *in vitro* models.

**Methods:**

Peripheral blood mononuclear cells (PBMC) from healthy donors were treated with PM10 and subsequently exposed to SARS-CoV-2 (D614G strain, MOI 0.1). The production of pro-inflammatory cytokines and antiviral factors was quantified by qPCR and ELISA. In addition, using the A549 cell line, previously exposed to PM, the viral replication was evaluated by qPCR and plaque assay.

**Results:**

SARS-CoV-2 stimulation increased the production of pro-inflammatory cytokines in PBMC, such as IL-1β, IL-6 and IL-8, but not antiviral factors. Likewise, PM10 induced significant production of IL-6 in PBMCs stimulated with SARS-CoV-2 and decreased the expression of OAS and PKR. Additionally, PM10 induces the release of IL-1β in PBMC exposed to SARS-CoV-2 as well as in a co-culture of epithelial cells and PBMCs. Finally, increased viral replication of SARS-CoV-2 was shown in response to PM10.

**Conclusion:**

Exposure to coarse particulate matter increases the production of pro-inflammatory cytokines, such as IL-1β and IL-6, and may alter the expression of antiviral factors, which are relevant for the immune response to SARS-CoV-2. These results suggest that pre-exposure to air particulate matter could have a modest role in the higher production of cytokines and viral replication during COVID-19, which eventually could contribute to severe clinical outcomes.

## 1 Introduction

COVID-19 is an acute respiratory illness caused by Severe Acute Respiratory Syndrome Coronavirus 2 (SARS-CoV-2), a pathogen that emerged in late 2019 and rapidly spread and became a global public health problem (1, 2). As of March 2023, SARS-CoV-2 has resulted in over 760 million infections and around 6.87 million deaths (3).

Although most individuals experience an asymptomatic or mild SARS-CoV-2 infection, some patients develop severe disease characterized by tissue damage and cytokine storm and may even develop acute respiratory distress syndrome (ARDS), which can lead to pulmonary failure and death (2). Severe forms of COVID-19 have been correlated with massive inflammatory cell infiltration and uncontrolled production of inflammatory mediators (2, 4); in fact, an association has been described between plasma IL-6 levels and hospitalization, intensive care unit (ICU) admission and mortality rates, suggesting that this cytokine may be a predictive factor of disease severity (5).

Risk factors contributing to severe forms of COVID-19 include older age, male gender (4), and comorbidities such as diabetes, hypertension and obesity (6). In addition, it has been suggested an association between exposure to environmental factors, such as air pollution, with the airborne transmission of SARS-CoV-2 and the severity of COVID-19 (7). In this regard, epidemiological studies carried out in three of the most affected areas by COVID-19, China, the USA, and Italy (8), have described an association between high levels of pollutants and increased morbidity and mortality (8–10).

Air pollution is another major public health problem in recent decades and has been associated with about 7 million deaths per year by the World Health Organization (WHO) (11). Among the primary pollutants is particulate matter (PM), a complex mixture of compounds derived from anthropogenic and natural sources. PM can be classified according to its aerodynamic diameter into coarse particles (with diameter between 10 and 2.5 µm; PM10), fine particles (with diameter < 2.5 µm; PM2.5) and ultrafine particles (with diameter < 0.1; PM0.1) (12). PM10 is mainly deposited in the upper respiratory tract and has been associated with diseases such as asthma (13), chronic obstructive pulmonary disease (COPD) (14), cardiovascular diseases (15, 16), neurologic diseases (17), tuberculosis (18) and cancer (19–21). An epidemiological study showed an association between increased lung cancer mortality (3.4 and 6%) and increased PM10 concentration by 10 µg/m^3^ (22).

In addition, previous studies have indicated that PM10 exposure is associated with increased incidence, viral transmissibility and severity of different respiratory viral infections (23), such as influenza A (24), measles (25), rhinovirus (26) and respiratory syncytial virus (RSV) (27–29). For example, an association between high levels of PM and a higher rate of hospitalization for bronchiolitis caused by RSV has been reported. Furthermore, PM concentrations have been correlated with epidemics caused by RNA viruses in recent decades, such as SARS-CoV in 2003 (30), dengue in 2007 (31), Influenza H1N1 in 2009 (32), Measles in 2019 (25) and recently the SARS-CoV-2 pandemic (10).

Although these associations have been described, the mechanisms by which PM contributes to the susceptibility or severity of respiratory viral infections are still unclear. Studies have described the ability of PM to induce oxidative stress and sustained inflammatory response, which promotes epithelial cell damage and cell recruitment to the lung; thus, contributing to lung tissue damage and impaired immune response to subsequent viral infections (33–36). Consistent with this, Xie et al. found that PM pre-exposure of Coxsackievirus-infected mice induced an increase lung and heart tissue damage, and an increase in cellular infiltrate (37). Likewise, it has been described that pre-exposure of mice to diesel particles increases susceptibility to influenza A infection, related to an increase in neutrophil recruitment and, IFN-β and IL-6 high levels (38). However, it is essential to consider factors such as the dose and time of exposure to PM on the effects that it can induce. In this regard, a study reported that chronic exposure to PM reduced IL-6 and IFN-β levels and increased Influenza A replication (33). Likewise, a study described that the immune response against high concentrations of PM10 and RSV, simultaneously, is less effective than the response against the virus alone, suggesting that exposure to PM alters the ability of cells to respond against viral infection, thus contributing to viral pathogenesis (39). Besides, it has been proposed that PM exposure predisposes towards the development of SARS-CoV-2-related immunopathology. However, the mechanisms involved in this relationship are still unclear; thus, this study aimed to evaluate the effect of PM on viral replication and the inflammatory response triggered by SARS-CoV-2.

## 2 Materials and methods

### 2.1 Cells and virus

Peripheral blood mononuclear cells (PBMC) were isolated by a Ficoll-histopaque density gradient method (Sigma-Aldrich Chemical Co., St. Louis, MO, USA) from each healthy donor (n = 3-7). The A549 cell line was grown in Dulbecco’s Modified Eagle Medium (DMEM, Sigma-Aldrich, St Louis, MO, USA) supplemented with 2% heat-inactivated fetal bovine serum (FBS) (GIBCO- Thermo Fisher Scientific Inc, Waltman, MA, USA), 2mM L-glutamine (Sigma-Aldrich Chemical Co., St. Louis, MO, USA) and 1% penicillin-streptomycin at 37°C with 5% CO_2_. The viral stock was produced from a Colombian SARS-CoV-2 isolated: D614G strain (EPI_ISL_536399) (40). The virus was used at 0.1 multiplicity of infection (MOI) and incubated for 8, 24 or 48h at 37°C with 5% CO_2_ (according to the experimental results). Unexposed cells were used as a negative control.

### 2.2 PM10 stock preparation

The PM10 was obtained through the local environmental agency (*Sistema de Alerta Temprana* - SIATA), a monitoring project established by the authority of the Valle de Aburrá - Colombia. Briefly, PM10 samples were obtained from quartz filters by sonication in deionized water. Then, the mixture was filtered and lyophilized. The working stock was prepared in sterile water at 10 mg/mL, sonicated and stored at -20°C until used.

### 2.3 PM10 and SARS-CoV-2 exposure in PBMC

The effect of PM10 in the inflammatory and antiviral response to SARS-CoV-2 was evaluated using a pre-exposure strategy. Briefly, PBMC were seeded (3x10^6^ cells/well) in RPMI-1640 supplemented with 5% FBS, exposed to PM10 (10 and 100 µg/mL) and incubated for 24 h at 37°C with 5% CO_2_. Then, the virus was added to wells and incubated for 24h. Supernatant and cells were harvested and stored at -70°C until processing. Untreated cells were used as a negative control. Seven independent experiments were conducted.

### 2.4 PM10 and SARS-CoV-2 exposure in co-culture of epithelial cell line and PBMC

The effect of PM10 in the production of IL-1β in a co-culture model of epithelial cell line and PBMC was evaluated. Briefly, A549 or VERO E6 cell line were seeded in DMEM supplemented with 2% FBS for 24 h at 37°C with 5% CO_2._ Then cells were exposed to PM10 (50 µg/mL for A549; 10 and 20 µg/mL for VERO E6) and incubated for 24 h at 37°C with 5% CO_2._ After incubation, supernatants were removed, and the virus (MOI 0.1 and 0.01, for A549 and VERO E6, respectively) was added and incubated at 37°C for 1h. PBMC freshly isolated were added to respective wells and incubated for 24h. The supernatant was harvested and stored at -70°C until processing. Untreated cells were used as a negative control. Three independent experiments were carried out.

Cytotoxic effect of PM10 on A549 and VERO E6 cells was evaluated using 3 -(4,5-dimethylthiazol-2-yl)-2,5-diphenyltetrazolium bromide (MTT) reduction assay, as previously reported (41). Briefly, cell lines were exposed to increasing concentrations of PM10 (1-400 µg/mL) for 48 h. The supernatant was removed and cells were incubated in fresh serum-free medium containing 0.5mg/mL MTT for 3h at 37°C in the dark. The formazan product was dissolved in DMSO and the absorbance was measured at 570nm using a microplate reader (Multiskan FC Microplate Photometer; Thermo Scientific). The data were normalized to the absorbance of the untreated control cells (**Supplementary Figure 1**).

### 2.5 Evaluation of the effect of PM10 in SARS-CoV-2 replication on A549 cells

A549 cells were cultured and exposed to PM10 for 24 h at 37°C with 5%CO_2_. After incubation, supernatants were removed, and the virus (MOI 0.1) was added and incubated at 37°C for 1h. Then, the virus inoculum was removed and DMEM (PM10 replenished) was added and incubated for 24h. The supernatant was harvested and stored at -70°C until processing. Infected cells without treatment were used as infection control. Cells without treatment nor infection were used as a negative control. Five independent experiments with two replicates each were performed.

### 2.6 RNA extraction, cDNA synthesis, and real-time PCR

The mRNA quantification of IL-1β, IL-6, IL-8, TNF-α, IFN-β, PKR (protein kinase R) and OAS (2'-5'-oligoadenylate synthetase 1) was carried out from PBMC by real-time PCR. Briefly, for total RNA extraction the Direct-zol RNA Miniprep kit was used (Zymo Research, Orange, CA, USA) following the manufacturer’s instructions. Then, RNA concentration and purity were determined by spectrophotometry at 260-280 nm and the cDNA was constructed using the iScript cDNA synthesis kit (BIO-RAD, Hercules, CA, USA). Real-time PCR was performed using Maxima SYBR Green qPCR master mix kit (Fermentas, Glen Burnie, MD, USA). The amplification protocols were standardized for each gene. PGK (phosphoglycerate kinase) was used as the housekeeping gene to normalize the RNA content; primer sequences for each gene are shown in **Supplementary Table 1**. The real-time PCR analysis was performed in the CFX Manager Version 1.5.534.511 software (Bio-Rad, Hercules, CA, USA). Data are expressed as fold change, normalized against the constitutive gene and the untreated control, using the ΔΔCt method, as previously reported (42).

### 2.7 Quantification of IL-1β, IL-6 and IL-8 by ELISA

Quantification of IL-1β, IL-6, and IL-8 levels in culture supernatants was carried out using ELISA kits (437004, Biolegend, Thermofisher; 555220, BD Biosciences, San Jose, CA, USA; and 431504 Biolegend, Thermofisher, respectively) as previously reported (42) and following manufacturer’s instructions.

### 2.8 Quantification of viral replication by qPCR and plaque assay

To quantify the viral RNA copies from supernatants, viral RNA extraction was performed using the commercial Quick-RNA Viral Kit (Zymo Research, Orange, CA) according to the manufacturer’s instructions. Viral RNA copies were quantified from 5uL of RNA by real time PCR using the Luna® Universal Probe One-Step RT-qPCR Kit (New England Biolabs) in the CFX-96 thermo-tracer (Biorad), following the Berlin protocol, version 2 (available at https://www.who.int/docs/default-source/coronaviruse/protocol-v2-1.pdf). Molecular degree water was used as a negative control and a plasmid that expresses each of the amplified genes (gene E, RdPp and RNASE P) were used as a positive control. The number of viral copies per mL was calculated using a standard curve of the plasmids containing the amplified genes (43).

The viral titer in supernatants was determined by plaque assay, as previously reported (44). Briefly, 1.2 x 10^5^ Vero E6 cells/well were seeded for 24h at 37°C, with 5% CO_2_. Then, 10-fold serial dilutions of the supernatants, obtained from assays in A549 cells, were added to cells and incubated for 1h. After incubation, the viral inoculum was removed and 1mL of 1.5% carboxymethyl-cellulose in DMEM 1X was added. Cells were incubated for 4 days at 37°C with 5% CO_2_, then washed twice with PBS and fixed/stained with 4% Formaldehyde/1% crystal violet solution; finally, the viral plaques were counted. The difference between viral titer of cells pre-exposed to PM10 and untreated control was expressed as an infection percentage.

### 2.9 Statistical analysis

GraphPad Prism 9.0.2 (La Jolla, CA, USA) was used for data analysis. Normality was assessed using Shapiro-Wilk test. ANOVA or Kruskal-Wallis tests were used to compare two or more groups; in case of statistical differences *post hoc* test (or multiple benchmarks) HDS of Tukey and Dunn, respectively, were applied. Data were presented as median ± IQR (Interquartile range).

### 2.10 Ethics

All experiments were carried out following the principles of the Declaration of Helsinki. Donors were adults, read and signed and informed consent, previously reviewed, and approved by the research ethics committee of the Universidad Cooperativa de Colombia.

## 3 Results

### 3.1 SARS-CoV-2 induces inflammatory cytokines in PBMC

**Figure 1:**
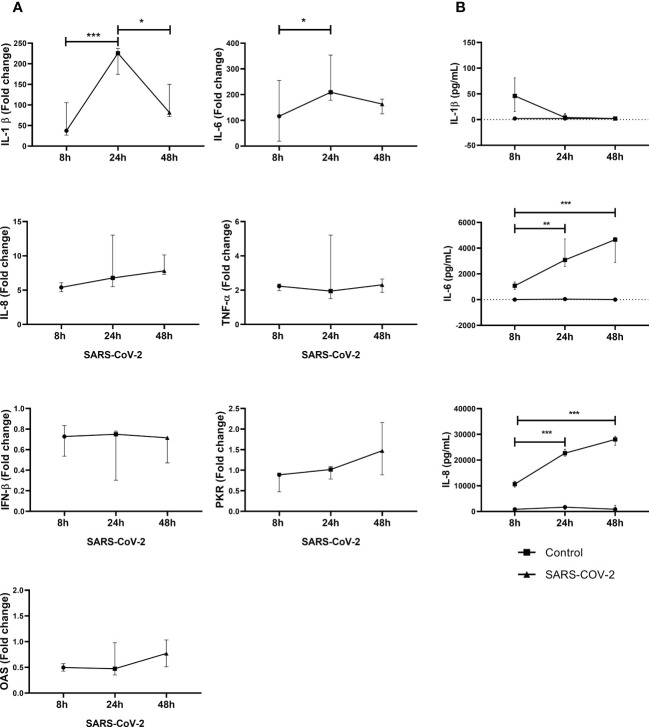
SARS-CoV-2 induces gene expression and secretion of inflammatory cytokines and antiviral factors in PBMC. Gene expression of inflammatory cytokines and antiviral factors was quantified by real-time PCR from PBMC exposed to SARS-CoV-2 (MOI 0.1). In addition, cytokine secretion in supernatants was evaluated by ELISA. Results of gene expression are presented as fold change of **(A)** IL-1βIL-6, IL-8, TNF-α, IFN-β, PKR and OAS compared with the unstimulated cells. Cytokine production is presented as pg/mL of **(B)** IL-1β, IL-6 and IL-8. Unexposed cells were used as a negative control. Data were represented as median ± IQR (n = 3 - biological replicates). Statistical comparison was made using the ANOVA or Kruskal-Wallis test, according to normality test, with a confidence level of 95% and *post hoc* tests (or multiple benchmarks) HDS of Tuckey or Dunn were applied. Significant differences *p ≤ 0.05, **p ≤ 0.01, ***p ≤ 0.001.

Increased IL-1β, IL-6, IL-8 and TNF-α mRNA expression were observed in PBMC exposed to SARS-CoV-2 compared to control cells (**Figure 1A**). Furthermore, the peak of increase in IL-1β and IL-6 was observed at 24 h. No statistical differences were observed for IL-8 and TNF-α during the experimental times.

In addition, SARS-CoV-2 also induced the secretion of IL-6 and IL-8 into cell culture supernatants. A significant increase in IL-6 and IL-8 was observed at 24h and 48h compared to 8h exposure (**Figure 1B**). In our experimental conditions, for IL-1β, a production peak was observed at 8h with a drop-in protein levels at 24h and 48h. Additionally, gene expression of common antivirals factors was evaluated. However, no changes in IFN-β, PKR and OAS (**Figure 1B**) were observed in response to SARS-CoV-2.

### 3.2 PM10 alters the expression of inflammatory cytokines and antiviral factors in SARS-CoV-2-exposed PMBC

**Figure 2:**
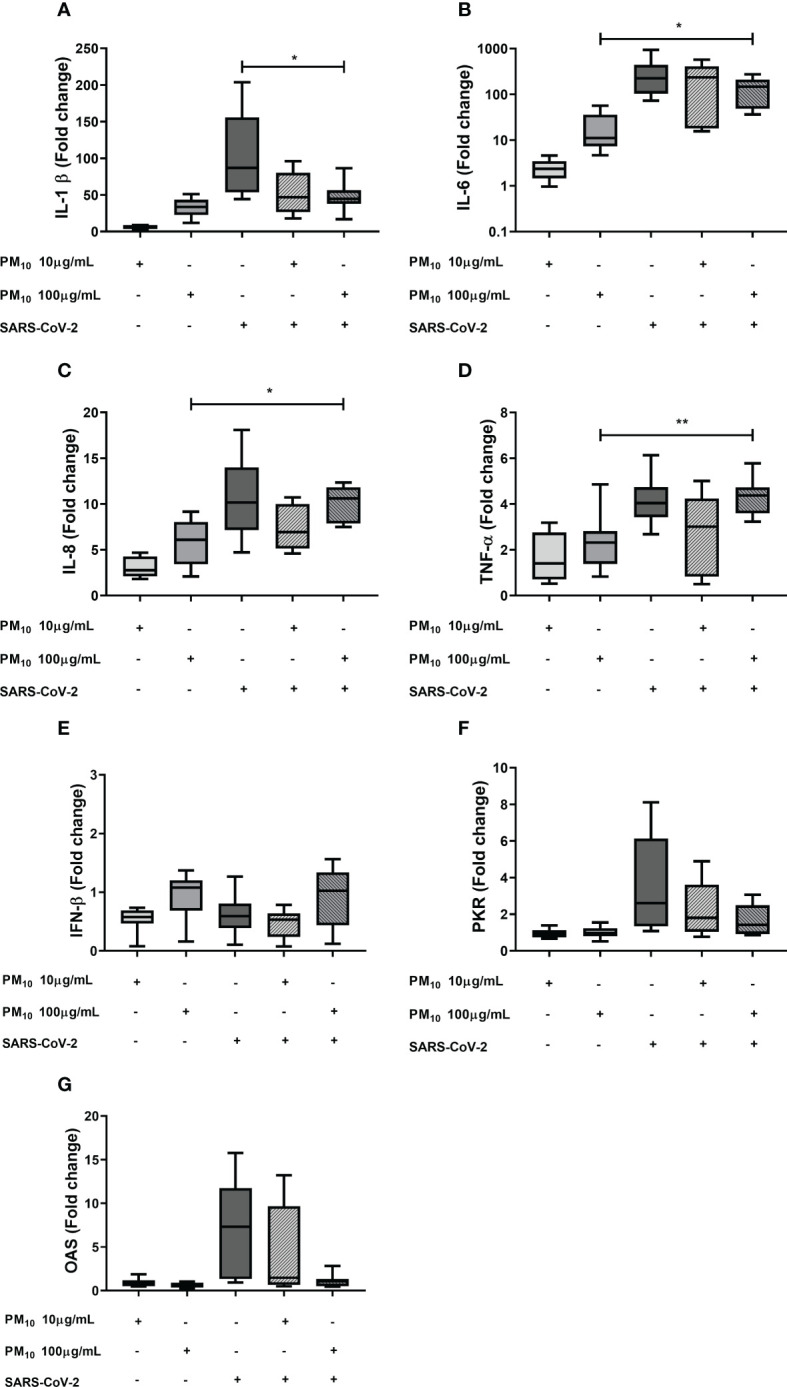
Effect of the PM10 in gene expression of inflammatory and antiviral factors in PBMC exposed to SARS-CoV-2. Gene expression of inflammatory and antiviral molecules was quantified by real-time PCR from PBMC exposed to PM10 and SARS-CoV-2 (MOI 0.1) after 48h of total treatment. Results are presented as fold change of **(A)** IL-1β, **(B)** IL-6, **(C)** IL-8, **(D)** TNF-α, **(E)** IFN-β, **(F)** PKR and **(G)** OAS. Cells unexposed were used as a negative control. Data were represented as median ± IQR (n = 7 - biological replicates). Statistical comparison was made using the Kruskal-Wallis test with a confidence level of 95% and *post hoc* tests (or multiple benchmarks) HDS of Dunn, were applied. Significant differences *p ≤ 0.05; **p<0.01.

The effect of previous exposure to PM10 on the gene expression of inflammatory and antiviral factors induced by SARS-CoV-2 was evaluated. A significative decrease in the fold change of IL-1β was observed in cells exposed to PM10 (100 µg/mL) and SARS-CoV-2 compared to cells exposed only to the virus. A significant difference in IL-6, IL-8 and TNF-α between cells co-stimulated with PM and SARS-CoV-2, compared to PM-treated cells, was observed. However, there were no significant differences in IL-6, IL-8, TNF-α and IFN-β when PBMC with both stimuli or only exposed to SARS-CoV-2 were compared (**Figure 2**). In addition, a trend towards the decreased expression of OAS and PKR was observed in PBMCs exposed to both stimuli compared to cells exposed to the virus alone; therefore, a statistical comparison was made only between these two groups, and it was found that there are statistically significant differences (**Supplementary Figure 2**).

### 3.3 PM10 increased the production of IL-6 in SARS-CoV-2-exposed PBMC

**Figure 3:**
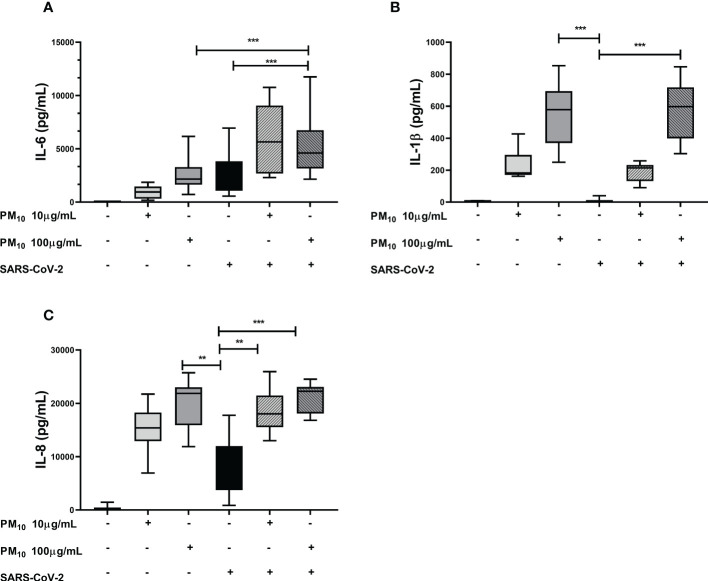
Effect of pre-exposure to PM10 in proinflammatory cytokine production in PBMC exposed to SARS-CoV-2. Production of inflammatory cytokines was quantified by ELISA from supernatants of PBMC exposed to PM10 and SARS-CoV-2 (MOI 0.1). Results are presented as pg/mL of **(A)** IL-6, **(B)** IL-1β y **(C)** IL-8. Cells unexposed were used as a negative control. Data were represented as median ± IQR (n = 7 - biological replicates). Statistical comparison was made using the Kruskal-Wallis test with a confidence level of 95% and *post hoc* tests (or multiple benchmarks) HDS of Dunn, were applied. Significant differences **p ≤ 0.01, ***p ≤ 0.001.

The IL-6 production was significantly increased in cells exposed to both PM10 and SARS-CoV-2 compared to cells individually exposed to PM10 (100µg/mL; p = 0.0006) or SARS-CoV-2 (p < 0.0001; **Figure 3A**). For IL-1β and IL-8, no additive effect was observed; however, the levels of these two cytokines were significantly higher in cells exposed to both PM10 (100µg/mL) and SARS-coV-2 compared to cells only exposed to SARS-CoV-2 (p < 0.0001) (**Figures 3B, C**). Likewise, no significant differences were observed in the production of IL-1β and IL-6 at PM10 concentrations of 20 and 50µg/mL. Although a trend towards an increase was observed with the 50μg/mL dose of PM10 for the production of both cytokines (**Supplementary Figure 3**).

### 3.4 PM10 induce IL-1β production in a SARS-CoV-2-exposed co-culture model of A549 and PBMC

**Figure 4:**
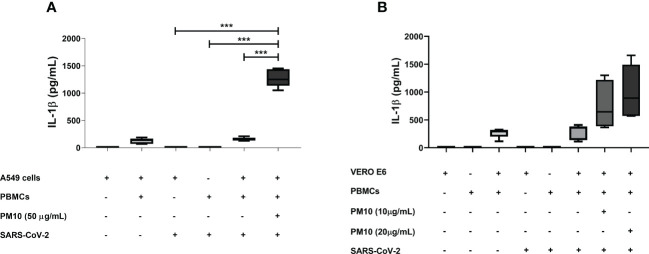
PM10 induce IL-1β production in a SARS-CoV-2-exposed co-culture model of A549 and PBMC. IL-1β production was quantified by ELISA from supernatants of co-culture of A549 or VERO E6 with PBMC and pre-exposure to PM10 and the infected with SARS-CoV-2. Results are presented as pg/mL of **(A)** co-culture A549 and PBMC, **(B)** VERO E6 and PBMC. Cells unexposed were used as a negative control. Data were represented as median ± IQR (n = 3 - biological replicates). Statistical comparison was made using the Kruskal-Wallis test with a confidence level of 95% and *post hoc* tests (or multiple benchmarks) HDS of Dunn, were applied. Significant differences ***p ≤ 0.001.

Significant production of IL-1β was observed in a co-culture model of A549 cells and PBMC exposed both to PM10 and SARS-CoV-2 compared to co-culture only exposed to SARS-CoV-2 (p < 0.0001; **Figure 4A**). Although no significant differences were found in the co-culture of VERO E6 and PBMCs, a trend toward an increase in the production of IL-1β was observed in co-culture exposed to both stimuli compared to co-culture exposed only to SARS-CoV-2 (**Figure 4B**). Therefore, a comparison was made only between these two groups, and it was found statistically significant differences (p = 0.0041; **Supplementary Figure 4**).

### 3.5 Pre-exposure to PM10 increases SARS-CoV-2 replication in A549 cells

**Figure 5:**
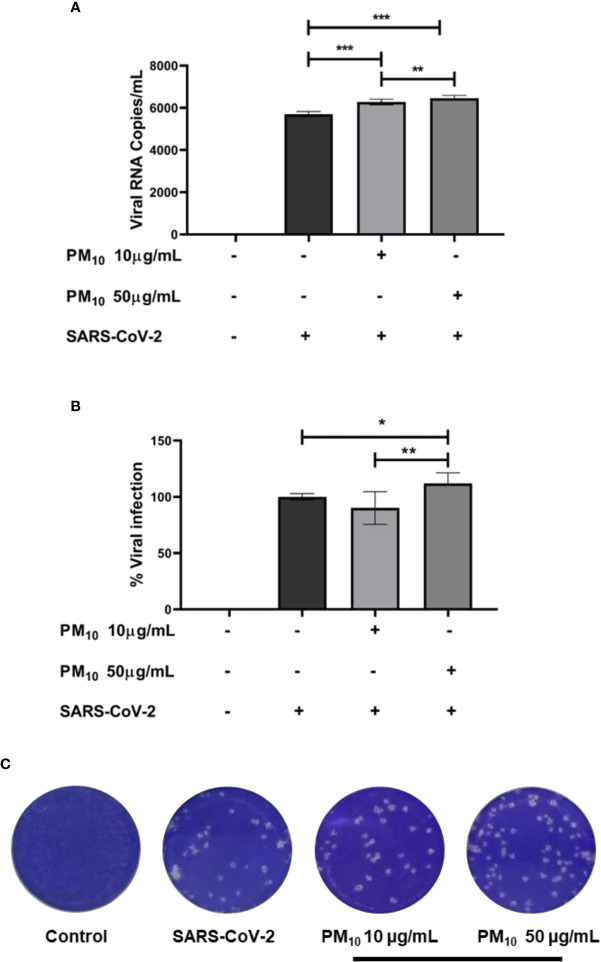
PM10 pre-exposure increase SARS-CoV-2 replication. Viral RNA copies and infectious virions were quantified by real time-PCR and plaque assay, respectively, from supernatants of A549 cells pre-exposed to PM10 and infected with SARS-CoV-2 (MOI 0.1). Results are presented as **(A)** viral RNA copies/mL, **(B)** percentage of infection from plaque assay, and **(C)** representative plaque assay. Infected cells and without treatment were used as infection control. Data were represented as median ± IQR (n = 7 - biological replicates). Statistical comparison was made using the Kruskal-Wallis test with a confidence level of 95% and *post hoc* tests (or multiple benchmarks) HDS of Dunn, were applied. Significant differences *p < 0.05, **p < 0.01, ***p < 0.001.

Finally, an increase in RNA viral copies of SARS-CoV-2 was observed in A549 cells pre-exposed to PM10 compared to infection control (p < 0.0001; **Figure 5A**). Furthermore, infectious viral particles were also increased in cells pre-exposed to 50µg/mL of PM10 (p = 0.0238; **Figures 5B, C**).

## 4 Discussion

Although most individuals experience asymptomatic or mild SARS-CoV-2 infections, some patients develop severe clinical conditions characterized by an exacerbated inflammatory response, known as a cytokine storm, which leads to tissue damage and multi-organ failure. In this study, we found that SARS-CoV-2 could induce an inflammatory response in PBMC, evidenced by the production of IL-1β, IL-6, IL-8 and TNF-α. Although respiratory epithelial cells are the main target cells of SARS-CoV-2, the acute immune response generated during infection is mediated mainly by immune cells, including (but not limited to) alveolar macrophages and monocyte-derived macrophages, in response to stimuli derived from infected cells (45). Monocytes from COVID-19 patients exhibited activated phenotype and increased production of pro-inflammatory cytokines compared with those from healthy individuals, indicating that these cells have an important role in the dysregulation of the inflammatory response during SARS-CoV-2 infection (46). Although many of the mechanisms involved in the development of the cytokine storm are still unknown, positive regulation of pro-inflammatory genes has been found in critical patients. This leads to recruitment of neutrophils and macrophages to the lung, thus increasing the cytokines and chemokines in BALF and blood and leading to a systemic inflammatory response with harmful effects in different tissues and organs.

In addition to the exacerbated production of inflammatory mediators, some studies have described a delayed antiviral response in COVID-19 patients that contributes to an unbalanced immune response and increased viral replication (47, 48). Our results did not show a significant increase in the production of antiviral molecules, even after 48 hours of stimulation. In general, the recognition of viral RNA by different intracellular pattern recognition receptors (PRRs) such as TLR3, TLR7/8, MDA5 or RIG-I leads to the production of IFN-I, inducing the transcription of ISGs (interferon-stimulated genes) which interfere with viral replication, constituting the primary antiviral mechanism. Several viruses have developed immune evasion strategies that counteract the IFN-I pathway, thus increasing viral fitness. In this regard, Scagnolari et al. found that patients requiring mechanical ventilation had a decreased expression of IFN-I and ISGs compared to those COVID-19 patients without ventilation requirements (48). In addition, an *ex vivo* study found that SARS-CoV-2 limits the response of interferons, despite its efficient replication in the lungs, suggesting the presence of immune evasion mechanisms, thus favoring viral transmissibility and pathogenesis (49). In line with this study, Yuen and colleagues found that SARS-CoV-2 proteins nsp13, nsp14, nsp15, and orf6 can suppress primary interferon production and its STAT-1-mediated signaling. In addition, neutralizing antibodies against IFN-I have been detected in patients with COVID-19, especially in those patients admitted to the ICU or with a fatal outcome (50–52). Taken together, peripheral blood cells contribute to the pathogenesis of SARS-CoV-2, through high production of pro-inflammatory cytokines and delayed antiviral response, which may lead to increased viral replication, which may ultimately trigger in the most severe forms of COVID-19.

Different risk factors have been described for developing severe forms of COVID-19, including environmental factors such as exposure to air pollutants. In this context, several studies have shown a relationship between air pollutants, such as PM, and susceptibility to respiratory viral infections, including those caused by RSV (28), rhinovirus (26), influenza A (24), measles (25), and coronaviruses (30). Although the mechanisms involved in the increased susceptibility and severity of viral infections remain unclear, it has been proposed that the oxidative and inflammatory response triggered by PM may contribute to viral pathogenesis. Likewise, it has been suggested that PM serves as a vehicle for virus transmission since viral RNA has been detected in PM samples; however, this hypothesis remains controversial and has not been proven (53). We found that cells previously exposed to PM10 have a higher production of IL-6 in response to SARS-CoV-2 compared to cells only exposed to the virus, suggesting that PM10 can potentiate the inflammatory response during COVID-19.

The increase in IL-6 production could contribute to the pathogenesis of SARS-CoV-2 infection, considering that critically ill patients show up to 10 times higher levels of IL-6 than patients with common disease (54). COVID-19 and these levels of IL-6 are positively correlated with the detection of viral RNA (55). In fact, patients who did not survive COVID-19 had higher levels of IL-6 than those who did survive (56). Likewise, Han et al. reported that critical COVID-19 patients had higher levels of IL-6 compared to those with moderate infections. In addition, a univariate logistic regression analysis, determined that IL-6 levels could be used as a predictor of severity (57).

Furthermore, this cytokine could be used as a predictor for mechanical ventilation requirement (58). On the other hand, Zhu et al. found a pathogenic Th1 response with high GM-CSF production in conjunction with highly inflammatory monocytes expressing high levels of IL-6 (59). It could be suggested that the significant increase in the production of inflammatory mediators such as IL-6, in response to the simultaneous stimulation of PM10 and SARS-CoV-2, may lead to an excessive immune response, accelerating the inflammatory process, greater recruitment of cells and favoring the tissue damage.

Interestingly, we observed that SARS-CoV-2 could induce IL-1β gene expression with a peak at 24h. However, when quantifying the cytokine in supernatant, we found moderate production at 8h and undetectable at 24h. Consistent with these results, Ma et al. reported that the SARS-CoV-2 nucleocapsid could interfere with the cleavage of Gasdermin D (60). This protein binds to phospholipids, forming pores in the membrane and triggering a form of death known as pyroptosis. When this process is inhibited, there is no release of the mature form of IL-1β, evidenced by lower production of IL-1β in infected monocytes (60). Moreover, when quantifying IL-1β in PBMC exposed to PM10, we found lower mRNA expression and higher cytokine release compared to those PBMC exposed only to the virus. Furthermore, a recent study shows that the interaction between epithelial cells and PBMC is necessary to induce IL-1β release in response to SARS-CoV-2 infection (61). In this sense, a significant increase in IL-1β production was found in the co-culture of A549 cells and PBMCs pre-exposed to PM10 and infected with SARS-CoV-2 compared with cells in co-culture but only exposed to SARS-CoV-2. Together, these findings indicate that, although the observed effect in cells exposed to both stimuli is due to PM10 exposure and not an additive effect, this PM-induced retained IL-1β release could contribute to the cytokine storm observed in the most critical patients. Furthermore, the production of IL-8 was higher in PBMC exposed to PM10 than exposed only to the SARS-CoV-2, suggesting that pre-exposure to PM can in general increased the production of pro-inflammatory cytokines and contribute to the imbalance inflammatory response observed in COVID-19 patients.

In contrast, we observed a decrease in PKR and OAS in cells exposed to SARS-CoV-2 and PM10 compared with PBMC exposed to virus. These results are according to previous reports of decrease IFN-β expression in cells treated with PM and then infected with the New-castle disease virus (NDV) and a highly pathogenic avian Influenza virus (H5N1) (62). This indicates that exposure to PM10 not only contributes to the inflammatory response but can also induce a dysregulation in the antiviral defense, leading to an inadequate immune response.

Finally, we found that A549 cells (epithelial cells from the respiratory tract) exposed to PM and infected with SARS-CoV-2 exhibit increased viral replication compared to control cells. Although PM exposure can induce an exacerbated inflammatory response that contributes to tissue damage, it can affect defense mechanisms in response to viral infections, contributing to viral susceptibility and pathogenesis. In this regard, Mishra et al. found that prior PM10 exposure of A549 cells prevented an adequate antiviral and inflammatory response against the H5N1 avian influenza virus, with increased viral RNA (62). According to the above, a study found an increase in the cytokine secretion and viral replication of Rhinovirus in human nasal epithelial cells exposed to sand dust (63). Similarly, an alteration of the early antiviral response was described in cells exposed to cigarette smoke and subsequently infected with Rhinovirus, leading to an increase in viral replication, which could explain how chronic exposure to air pollutants contributes to greater susceptibility to viral infections (64). Additionally, other factors could contribute to the effects of air pollutants. In this sense, Ural et al. found a specifically age-related decline in the immune function of lung-associated lymph nodes, which is linked with the accumulation of particulate matter in them (65). These results suggest that older people, chronically exposed to PM, are more likely to develop an altered and less effective immune response against viral infections such as that caused by SARS-CoV-2. Thus, increasing the risk of developing severe forms of the disease than younger people.

Our approach has limitations, including the use of cell lines, which are great tools for *in vitro* evaluations; however, their carcinogenic phenotype could be masking some of the effects of PM. In addition, the pseudo-stratification of the respiratory epithelium could also be contributing to differential response. For this reason, it would be important to carry out studies using primary respiratory epithelial cells under culture conditions that allow their stratification and evaluate how PM and SARS-CoV-2 infection interact in the different cells. In addition, the exposure time to PM triggers different adverse effects; accordingly, it would be appropriate to carry out new experiments where the change in the response over time is evaluated. Lastly, differences in the immune response related to the age of people have been described. Likewise, a risk factor described for the development of severe forms of COVID-19 is age. In accordance with this, the evaluation of primary lung epithelial cells in different age donors could provide information on whether there is an addictive effect between PM and SARS-CoV-2 in older people.

## 5 Conclusion

In summary, our results suggest that previous exposure to coarse PM induces a modest increase in the production of inflammatory mediators, such as IL-1β and IL-6 and may alter the expression of interferon response genes. These alterations in the immune response could favor viral replication and thus contribute to the pathogenesis of COVID-19. However, our *in vitro* model shows minor changes, being necessary to perform new experiments that confirm the role of PM pre-exposure in the development of the severe forms of COVID-19.

## Data availability statement

The original contributions presented in the study are included in the article/**Supplementary Material**. Further inquiries can be directed to the corresponding author.

## Ethics statement

The studies involving human participants were reviewed and approved by Ethics committee Universidad Cooperativa de Colombia. The patients/participants provided their written informed consent to participate in this study. Informed consent was obtained from all subjects involved in the study.

## Author contributions

Conceptualization: DM-P, NT, MTR, and JCH; Writing - Original Draft: DM-P and JCH; Writing - Review and Editing: JHT-G, MIZ-C, WZ-B, NT, and MTR; Investigation: DM-P, JHT-G, and MIZ-C; Methodology: DM-P, WZ-B, and JCH; Visualization: DM-P; Formal analysis: DM-P, NT and JCH; Project administration: NT and JCH.

## References

[1] Park SE. Epidemiology, virology, and clinical features of severe acute respiratory syndrome -coronavirus-2 (SARS-CoV-2; coronavirus disease-19). Clin Exp Pediatr (2020) 63(4):119–24. doi: 10.3345/cep.2020.00493 PMC7170784 32252141

[2] Huang C Wang Y Li X Ren L Zhao J Hu Y. Clinical features of patients infected with 2019 novel coronavirus in wuhan, China. Lancet (2020) 395(10223):497–506. doi: 10.1016/S0140-6736(20)30183-5 31986264 PMC7159299

[3] WHO. WHO coronavirus (COVID-19) dashboard 2022 (2022). Available at: https://covid19.who.int/.

[4] Wu C Chen X Cai Y Xia J Zhou X Xu S. Risk factors associated with acute respiratory distress syndrome and death in patients with coronavirus disease 2019 pneumonia in wuhan, China. JAMA Intern Med (2020) 180(7):934–43. doi: 10.1001/jamainternmed.2020.0994 PMC7070509 32167524

[5] Aziz M Fatima R Assaly R. Elevated interleukin-6 and severe COVID-19: a meta-analysis. J Med Virol (2020) 92(11):2283–5. doi: 10.1002/jmv.25948 PMC7267383 32343429

[6] Gao YD Ding M Dong X Zhang JJ Kursat Azkur A Azkur D. Risk factors for severe and critically ill COVID-19 patients: a review. Allergy (2021) 76(2):428–55. doi: 10.1111/all.14657 33185910

[7] Copat C Cristaldi A Fiore M Grasso A Zuccarello P Signorelli SS. The role of air pollution (PM and NO2) in COVID-19 spread and lethality: a systematic review. Environ Res (2020) 191:110129. doi: 10.1016/j.envres.2020.110129 32853663 PMC7444490

[8] Fattorini D Regoli F. Role of the chronic air pollution levels in the covid-19 outbreak risk in Italy. Environ pollut (2020) 264:114732. doi: 10.1016/j.envpol.2020.114732 32387671 PMC7198142

[9] Wu X Nethery RC Sabath BM Braun D Dominici F. Exposure to air pollution and COVID-19 mortality in the united states: a nationwide cross-sectional study. Sci Adv (2020) 6(45):eabd4049. doi: 10.1101/2020.04.05.20054502 PMC7673673 33148655

[10] Yao Y Pan J Liu Z Meng X Wang W Kan H. Temporal association between particulate matter pollution and case fatality rate of COVID-19 in wuhan. Environ Res (2020) 189:109941. doi: 10.1016/j.envres.2020.109941 32678728 PMC7361083

[11] Collaborators GBDRF. Global, regional, and national comparative risk assessment of 84 behavioural, environmental and occupational, and metabolic risks or clusters of risks for 195 countries and territories, 1990-2017: a systematic analysis for the global burden of disease study 2017. Lancet (2018) 392(10159):1923–94. doi: 10.1016/S0140-6736(18)32225-6 PMC6227755 30496105

[12] Jiang SY Gali NK Yang F Zhang J Ning Z. Chemical characterization of size-segregated PM from different public transport modes and implications of source specific contribution to public exposure. Environ Sci pollut Res Int (2017) 24(24):20029–40. doi: 10.1007/s11356-017-9661-6 28699010

[13] Tecer LH Alagha O Karaca F Tuncel G Eldes N. Particulate matter (PM(2.5), PM(10-2.5), and PM(10)) and children’s hospital admissions for asthma and respiratory diseases: a bidirectional case-crossover study. J Toxicol Environ Health A (2008) 71(8):512–20. doi: 10.1080/15287390801907459 18338286

[14] Doiron D de Hoogh K Probst-Hensch N Fortier I Cai Y De Matteis S. Air pollution, lung function and COPD: results from the population-based UK biobank study. Eur Respir J (2019) 54(1):1802140. doi: 10.1183/13993003.02140-2018 31285306

[15] Nasser Z Salameh P Nasser W Abou Abbas L Elias E Leveque A. Outdoor particulate matter (PM) and associated cardiovascular diseases in the middle East. Int J Occup Med Environ Health (2015) 28(4):641–61. doi: 10.13075/ijomeh.1896.00186 26216305

[16] Takeuchi A Nishiwaki Y Okamura T Milojevic A Ueda K Asakura K. Long-term exposure to particulate matter and mortality from cardiovascular diseases in Japan: the ibaraki prefectural health study (IPHS). J Atheroscler Thromb (2021) 28(3):230–40. doi: 10.5551/jat.54148 PMC8048949 32641588

[17] Heusinkveld HJ Wahle T Campbell A Westerink RHS Tran L Johnston H. Neurodegenerative and neurological disorders by small inhaled particles. Neurotoxicology (2016) 56:94–106. doi: 10.1016/j.neuro.2016.07.007 27448464

[18] Sarkar S Rivas-Santiago CE Ibironke OA Carranza C Meng Q Osornio-Vargas A. Season and size of urban particulate matter differentially affect cytotoxicity and human immune responses to mycobacterium tuberculosis. PloS One (2019) 14(7):e0219122. doi: 10.1371/journal.pone.0219122 31295271 PMC6622489

[19] Consonni D Carugno M De Matteis S Nordio F Randi G Bazzano M. Outdoor particulate matter (PM10) exposure and lung cancer risk in the EAGLE study. PloS One (2018) 13(9):e0203539. doi: 10.1371/journal.pone.0203539 30216350 PMC6157824

[20] Marin-Palma D González JD Narváez J Porras J Taborda NA Hernandez JC. Physicochemical characterization and evaluation of the cytotoxic effect of particulate matter (PM10). Water Air Soil Pollut (2023) 234:138. doi: 10.1007/s11270-023-06155-5

[21] Arias-Perez RD Taborda NA Gomez DM Narvaez JF Porras J Hernandez JC. Inflammatory effects of particulate matter air pollution. Environ Sci pollut Res Int (2020) 27(34):42390–404. doi: 10.1007/s11356-020-10574-w 32870429

[22] Chen X Zhang LW Huang JJ Song FJ Zhang LP Qian ZM. Long-term exposure to urban air pollution and lung cancer mortality: a 12-year cohort study in northern China. Sci Total Environ (2016) 571:855–61. doi: 10.1016/j.scitotenv.2016.07.064 27425436

[23] Hei Collaborative Working Group on Air Pollution P Health in Ho Chi Minh C TG L Ngo L Mehta S VD D. Effects of short-term exposure to air pollution on hospital admissions of young children for acute lower respiratory infections in ho chi minh city, Vietnam. Res Rep Health Eff Inst (2012) 169):5–72. 22849236

[24] Chen G Zhang W Li S Zhang Y Williams G Huxley R. The impact of ambient fine particles on influenza transmission and the modification effects of temperature in China: a multi-city study. Environ Int (2017) 98:82–8. doi: 10.1016/j.envint.2016.10.004 PMC7112570 27745688

[25] Peng L Zhao X Tao Y Mi S Huang J Zhang Q. The effects of air pollution and meteorological factors on measles cases in lanzhou, China. Environ Sci pollut Res Int (2020) 27(12):13524–33. doi: 10.1007/s11356-020-07903-4 32030582

[26] Vempilly J Abejie B Diep V Gushiken M Rawat M Tyner TR. The synergetic effect of ambient PM2.5 exposure and rhinovirus infection in airway dysfunction in asthma: a pilot observational study from the central valley of California. Exp Lung Res (2013) 39(10):434–40. doi: 10.3109/01902148.2013.840693 24245976

[27] Karr CJ Rudra CB Miller KA Gould TR Larson T Sathyanarayana S. Infant exposure to fine particulate matter and traffic and risk of hospitalization for RSV bronchiolitis in a region with lower ambient air pollution. Environ Res (2009) 109(3):321–7. doi: 10.1016/j.envres.2008.11.006 PMC2925442 19211100

[28] Nenna R Evangelisti M Frassanito A Scagnolari C Pierangeli A Antonelli G. Respiratory syncytial virus bronchiolitis, weather conditions and air pollution in an Italian urban area: an observational study. Environ Res (2017) 158:188–93. doi: 10.1016/j.envres.2017.06.014 PMC7125886 28647513

[29] Loaiza-Ceballos MC Marin-Palma D Zapata W Hernandez JC. Viral respiratory infections and air pollutants. Air Qual Atmos Health (2022) 15(1):105–14. doi: 10.1007/s11869-021-01088-6 PMC8441953 34539932

[30] Cui Y Zhang ZF Froines J Zhao J Wang H Yu SZ. Air pollution and case fatality of SARS in the people’s republic of China: an ecologic study. Environ Health (2003) 2(1):15. doi: 10.1186/1476-069X-2-15 14629774 PMC293432

[31] Massad E Coutinho FA Ma S Burattini MN. A hypothesis for the 2007 dengue outbreak in Singapore. Epidemiol Infect (2010) 138(7):951–7. doi: 10.1017/S0950268809990501 19653928

[32] Morales KF Paget J Spreeuwenberg P. Possible explanations for why some countries were harder hit by the pandemic influenza virus in 2009 - a global mortality impact modeling study. BMC Infect Dis (2017) 17(1):642. doi: 10.1186/s12879-017-2730-0 28946870 PMC5613504

[33] Cevallos VM Diaz V Sirois CM. Particulate matter air pollution from the city of Quito, Ecuador, activates inflammatory signaling pathways *in vitro* . Innate Immun (2017) 23(4):392–400. doi: 10.1177/1753425917699864 28409539

[34] Piao MJ Ahn MJ Kang KA Ryu YS Hyun YJ Shilnikova K. Particulate matter 2.5 damages skin cells by inducing oxidative stress, subcellular organelle dysfunction, and apoptosis. Arch Toxicol (2018) 92(6):2077–91. doi: 10.1007/s00204-018-2197-9 PMC6002468 29582092

[35] Zosky GR Iosifidis T Perks K Ditcham WG Devadason SG Siah WS. The concentration of iron in real-world geogenic PM(1)(0) is associated with increased inflammation and deficits in lung function in mice. PloS One (2014) 9(2):e90609. doi: 10.1371/journal.pone.0090609 24587402 PMC3938778

[36] Valderrama A Ortiz-Hernandez P Agraz-Cibrian JM Tabares-Guevara JH Gomez DM Zambrano-Zaragoza JF. Particulate matter (PM10) induces *in vitro* activation of human neutrophils, and lung histopathological alterations in a mouse model. Sci Rep (2022) 12(1):7581. doi: 10.1038/s41598-022-11553-6 35534522 PMC9083477

[37] Xie Y Zhang X Tian Z Jiang R Chen R Song W. Preexposure to PM2.5 exacerbates acute viral myocarditis associated with Th17 cell. Int J Cardiol (2013) 168(4):3837–45. doi: 10.1016/j.ijcard.2013.06.025 23849969

[38] Ciencewicki J Gowdy K Krantz QT Linak WP Brighton L Gilmour MI. Diesel exhaust enhanced susceptibility to influenza infection is associated with decreased surfactant protein expression. Inhal Toxicol (2007) 19(14):1121–33. doi: 10.1080/08958370701665426 17987464

[39] Becker S Soukup JM. Exposure to urban air particulates alters the macrophage-mediated inflammatory response to respiratory viral infection. J Toxicol Environ Health A (1999) 57(7):445–57. doi: 10.1080/009841099157539 10494914

[40] Diaz FJ Aguilar-Jimenez W Florez-Alvarez L Valencia G Laiton-Donato K Franco-Munoz C. Isolation and characterization of an early SARS-CoV-2 isolate from the 2020 epidemic in medellin, Colombia. Biomedica (2020) 40(Supl. 2):148–58. doi: 10.7705/biomedica.5834 PMC7676823 33152198

[41] Marin-Palma D González JD Narváez JF Porras J Taborda NA Hernandez JC. Physicochemical characterization and evaluation of the cytotoxic effect of particulate matter (PM10). Water Air Soil Pollution (2023) 234(3):138. doi: 10.1007/s11270-023-06155-5

[42] Marin-Palma D Tabares-Guevara JH Zapata-Cardona MI Florez-Alvarez L Yepes LM Rugeles MT. Curcumin inhibits *In Vitro* SARS-CoV-2 infection in vero E6 cells through multiple antiviral mechanisms. Molecules (2021) 26(22):6900. doi: 10.3390/molecules26226900 PMC8618354 34833991

[43] Zapata-Cardona MI Florez-Alvarez L Gómez D Moncada-Díaz J Hernandez J Diaz F. Comparison among plaque assay, tissue culture infectious dose (TCID50) and real-time RT-PCR for SARS-CoV-2 variants quantification. Iranian J Microbiol (2022) 14(3):20–40. doi: 10.18502/ijm.v14i3.9758 PMC10132340 37124861

[44] Zapata-Cardona MI Florez-Alvarez L Zapata-Builes W Guerra-Sandoval AL Guerra-Almonacid CM Hincapie-Garcia J. Atorvastatin effectively inhibits ancestral and two emerging variants of SARS-CoV-2 *in vitro* . Front Microbiol (2022) 13:721103. doi: 10.3389/fmicb.2022.721103 35369500 PMC8972052

[45] Liao M Liu Y Yuan J Wen Y Xu G Zhao J. Single-cell landscape of bronchoalveolar immune cells in patients with COVID-19. Nat Med (2020) 26(6):842–4. doi: 10.1038/s41591-020-0901-9 32398875

[46] Zhang D Guo R Lei L Liu H Wang Y Wang Y. Frontline science: COVID-19 infection induces readily detectable morphologic and inflammation-related phenotypic changes in peripheral blood monocytes. J Leukoc Biol (2021) 109(1):13–22. doi: 10.1002/JLB.4HI0720-470R 33040384 PMC7675546

[47] Blanco-Melo D Nilsson-Payant BE Liu WC Uhl S Hoagland D Moller R. Imbalanced host response to SARS-CoV-2 drives development of COVID-19. Cell (2020) 181(5):1036–45 e9. doi: 10.1016/j.cell.2020.04.026 32416070 PMC7227586

[48] Scagnolari C Pierangeli A Frasca F Bitossi C Viscido A Oliveto G. Differential induction of type I and III interferon genes in the upper respiratory tract of patients with coronavirus disease 2019 (COVID-19). Virus Res (2021) 295:198283. doi: 10.1016/j.virusres.2020.198283 33418027 PMC7834390

[49] Chu H Chan JF Wang Y Yuen TT Chai Y Hou Y. Comparative replication and immune activation profiles of SARS-CoV-2 and SARS-CoV in human lungs: an ex vivo study with implications for the pathogenesis of COVID-19. Clin Infect Dis (2020) 71(6):1400–9. doi: 10.1093/cid/ciaa410 PMC7184390 32270184

[50] Chen K Xiao F Hu D Ge W Tian M Wang W. SARS-CoV-2 nucleocapsid protein interacts with RIG-I and represses RIG-mediated IFN-beta production. Viruses (2020) 13(1):47. doi: 10.3390/v13010047 PMC7823417 33396605

[51] Frasca F Scordio M Santinelli L Gabriele L Gandini O Criniti A. Anti-IFN-alpha/-omega neutralizing antibodies from COVID-19 patients correlate with downregulation of IFN response and laboratory biomarkers of disease severity. Eur J Immunol (2022) 52(7):1120–8. doi: 10.1002/eji.202249824 PMC9087404 35419822

[52] Yuen CK Lam JY Wong WM Mak LF Wang X Chu H. SARS-CoV-2 nsp13, nsp14, nsp15 and orf6 function as potent interferon antagonists. Emerg Microbes Infect (2020) 9(1):1418–28. doi: 10.1080/22221751.2020.1780953 PMC7473193 32529952

[53] Setti L Passarini F De Gennaro G Barbieri P Perrone MG Borelli M. SARS-Cov-2RNA found on particulate matter of bergamo in northern Italy: first evidence. Environ Res (2020) 188:109754. doi: 10.1016/j.envres.2020.109754 32526492 PMC7260575

[54] Zhang J Hao Y Ou W Ming F Liang G Qian Y. Serum interleukin-6 is an indicator for severity in 901 patients with SARS-CoV-2 infection: a cohort study. J Transl Med (2020) 18(1):406. doi: 10.1186/s12967-020-02571-x 33121497 PMC7594951

[55] Chen X Zhao B Qu Y Chen Y Xiong J Feng Y. Detectable serum severe acute respiratory syndrome coronavirus 2 viral load (RNAemia) is closely correlated with drastically elevated interleukin 6 level in critically ill patients with coronavirus disease 2019. Clin Infect Dis (2020) 71(8):1937–42. doi: 10.1093/cid/ciaa449 PMC7184354 32301997

[56] Avila-Nava A Cortes-Telles A Torres-Erazo D Lopez-Romero S Chim Ake R Gutierrez Solis AL. Serum IL-6: a potential biomarker of mortality among SARS-CoV-2 infected patients in Mexico. Cytokine (2021) 143:155543. doi: 10.1016/j.cyto.2021.155543 33896708 PMC8052471

[57] Han H Ma Q Li C Liu R Zhao L Wang W. Profiling serum cytokines in COVID-19 patients reveals IL-6 and IL-10 are disease severity predictors. Emerg Microbes Infect (2020) 9(1):1123–30. doi: 10.1080/22221751.2020.1770129 PMC7473317 32475230

[58] Herold T Jurinovic V Arnreich C Lipworth BJ Hellmuth JC von Bergwelt-Baildon M. Elevated levels of IL-6 and CRP predict the need for mechanical ventilation in COVID-19. J Allergy Clin Immunol (2020) 146(1):128–36 e4. doi: 10.1016/j.jaci.2020.05.008 32425269 PMC7233239

[59] Zhou Y Fu B Zheng X Wang D Zhao C Qi Y. Pathogenic T-cells and inflammatory monocytes incite inflammatory storms in severe COVID-19 patients. Natl Sci Rev (2020) 7(6):998–1002. doi: 10.1093/nsr/nwaa041 34676125 PMC7108005

[60] Ma J Zhu F Zhao M Shao F Yu D Ma J. SARS-CoV-2 nucleocapsid suppresses host pyroptosis by blocking gasdermin d cleavage. EMBO J (2021) 40(18):e108249. doi: 10.15252/embj.2021108249 34296442 PMC8420271

[61] Barnett KC Xie Y Asakura T Song D Liang K Taft-Benz SA. An epithelial-immune circuit amplifies inflammasome and IL-6 responses to SARS-CoV-2. Cell Host Microbe (2023) 31(2):243–59 e6. doi: 10.1016/j.chom.2022.12.005 36563691 PMC9731922

[62] Mishra R Krishnamoorthy P Gangamma S Raut AA Kumar H. Particulate matter (PM10) enhances RNA virus infection through modulation of innate immune responses. Environ pollut (2020) 266(1):115148. doi: 10.1016/j.envpol.2020.115148 PMC7357538 32771845

[63] Yeo NK Hwang YJ Kim ST Kwon HJ Jang YJ. Asian Sand dust enhances rhinovirus-induced cytokine secretion and viral replication in human nasal epithelial cells. Inhal Toxicol (2010) 22(12):1038–45. doi: 10.3109/08958378.2010.516282 20879958

[64] Eddleston J Lee RU Doerner AM Herschbach J Zuraw BL. Cigarette smoke decreases innate responses of epithelial cells to rhinovirus infection. Am J Respir Cell Mol Biol (2011) 44(1):118–26. doi: 10.1165/rcmb.2009-0266OC PMC3028255 20224072

[65] Ural BB Caron DP Dogra P Wells SB Szabo PA Granot T. Inhaled particulate accumulation with age impairs immune function and architecture in human lung lymph nodes. Nat Med (2022) 28(12):2622–32. doi: 10.1038/s41591-022-02073-x PMC9835154 36411343

