# Supplementary material for: PM10 promotes an inflammatory cytokine response that may impact SARS-CoV-2 replication in vitro
Source: Front Immunol. 2023 Apr 25;14:1161135. doi: 10.3389/fimmu.2023.1161135 (PMC10166799; doi:10.3389/fimmu.2023.1161135)
Supplement: Supplementary file 1 [file DataSheet_1.docx]

Supplementary Material

**PM10 promotes an inflammatory cytokine response that may impact SARS-CoV-2 replication *in vitro***

**Damariz Marín-Palma^1,2^, Jorge H. Tabares-Guevara^2^, María I. Zapata-Cardona^2^, Wildeman Zapata-Builes^1,2^, Natalia Taborda^2,3^, Maria T. Rugeles^2^, Juan C. Hernandez^1,2*^**

*** Correspondence:** Corresponding Author: (Hernandez, JC) juankhernandez@gmail.com

# Supplementary Figures and Tables

## Supplementary Figures

**Supplementary Figure 1. PM10 effects on viability on A549 and VERO E6 cells.** A549 and VERO E6 cells were exposed to increasing doses of PM10 for 48 h, using as a positive control DMSO. Data were represented as median ± IQR (n =3.) Statistical comparison was made using the Kruskal-Wallis test with a confidence level of 95% and post hoc tests (or multiple benchmarks) HDS of Dunn, were applied. Significant differences **p <0.01 and *** p < 0.001.


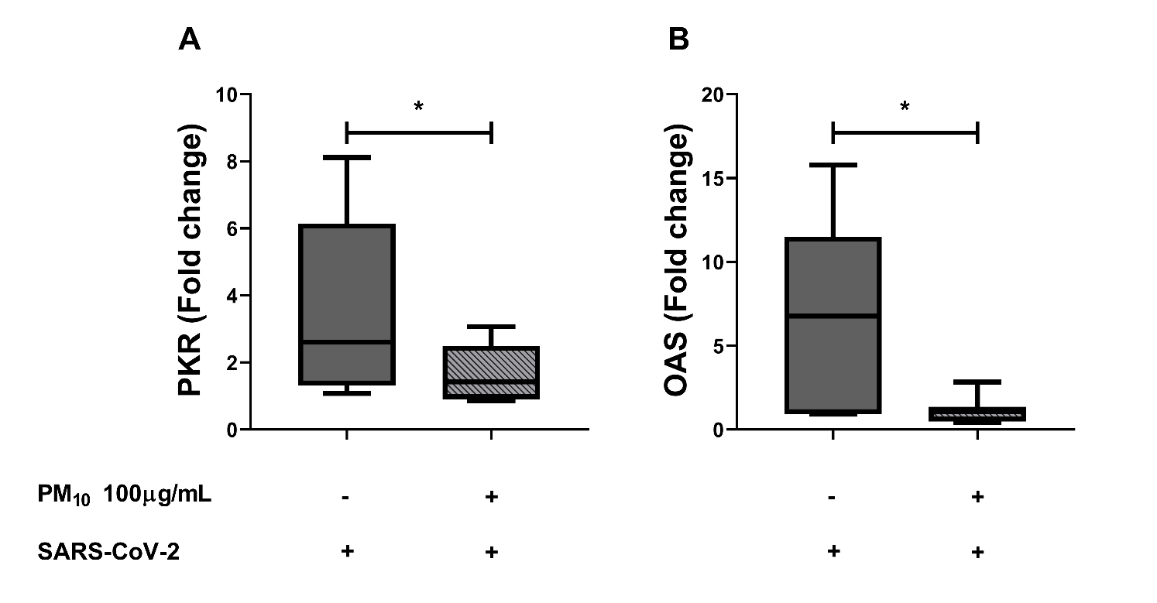


**Supplementary Figure 2. Effect of the PM10 in gene expression PKR and OAS in PBMC exposed to SARS-CoV-2**. Gene expression PKR and OAS was quantified by real-time PCR from PBMC exposed to PM10 and SARS-CoV-2 (MOI 0.1). Results are presented as fold change of **(A)** PKR, **(B)** OAS. Data were represented as median ± IQR (n =7). Statistical comparison was made using the Mann-Whitney test with a confidence level of 95%. Significant differences *p ≤ 0.05.

**Supplementary Figure 3. Effect of pre-exposure to PM10 in proinflammatory cytokine production in PBMC exposed to SARS-CoV-2.** Production of inflammatory cytokines was quantified by ELISA from supernatants of PBMC exposed to PM10 and SARS-CoV-2 (MOI 0.1). Results are presented as pg/mL of (A) IL-1β and (B) IL-6. Cells unexposed were used as a negative control. Data were represented as median ± IQR (n =3). Statistical comparison was made using the Kruskal-Wallis test with a confidence level of 95% and post hoc tests (or multiple benchmarks) HDS of Dunn, were applied.

**Supplementary Figure 4. PM10 induces IL-1β production in a SARS-CoV-2-exposed co-culture model of VERO E6 and PBMC.** IL-1β production was quantified by ELISA from supernatants of co-culture VERO E6 with PBMC and pre-exposure to PM10 and the infected with SARS-CoV-2. Results are presented as pg/mL. Data were represented as median ± IQR (n =3). Statistical comparison was made using the Mann-Whitney test with a confidence level of 95%. Significant differences* *p ≤ 0.01.

## Supplementary Tables

**Supplementary Table 1. Primer sequences**

| **Gene** | **Primers 5´-3´** | ***Annealing temperature*** |
| --- | --- | --- |
| **IL-1β** | Fw: GGATATGGAGCAACAAGTGG  Rv: ATGTACCAGTTGGGGAACTG | 60 °C |
| **IL-6** | Fw: GGGGTGGTTATTGCATC  Rv: ATTCGGTACATCCTCGAC | 56 °C |
| **IL-8** | Fw: ACTGAGAGTGATTGAGAGTGGAC  Rv: AACCCTCTGCACCCAGTTTTC | 60 °C |
| **TNF-α** | Fw: GGCTCCAGGCGGTGCTTGTTC  Rv: AGACGGCGATGCGGCTGATG | 60 °C |
| **IFN-β** | Fw: ATGACCAACAAGTGTCTCCTCC  Rv: GCTCATGGAAAGAGCTGTAGTG | 60 °C |
| **PKR** | Fw: TCTTCATGTATGTGACACTGC  Rv: CACACAGTCAAGGTCCTTAG | 60 °C |
| **OAS** | Fw: GTGTGTCCAAGGTGGTAAAGG  Rv: CTGCTCAAACTTCACGGAA | 60 °C |
| **PGK** | Fw: GTTGACCGAATCACCGACC Rv: CGACTCTCATAACGACCCGC | 60 °C |
